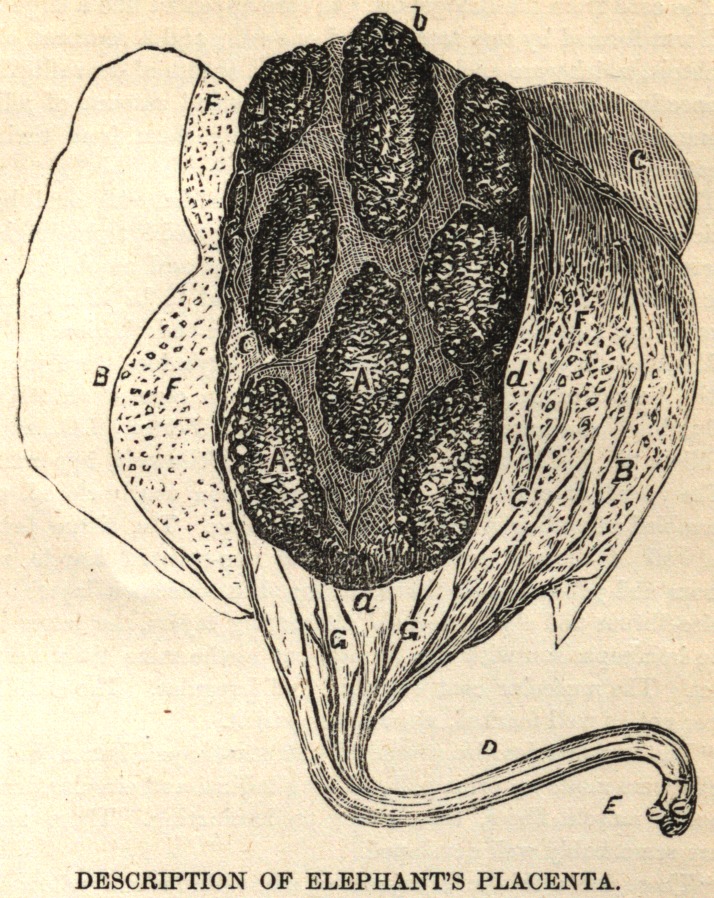# Breeding of Elephants in Captivity

**Published:** 1882-04

**Authors:** George Arstingstall

**Affiliations:** Elephant Trainer for Barnum, Bailey and Hutchinson


### ART. XIII.—BREEDING OF ELEPHANTS IN CAPTIVITY.

BY GEORGE ARSTINGSTALL,

Elephant Trainer for Barnum, Bailey and Hutchinson.

The breeding of elephants in captivity is not very common. Elephants bred in captivity are spoken of by Œlian even in the days of ancient Rome; but from that day to this it has been a rare occurrence, even in India and Ceylon. In Ava, however, it is quite a common thing, as the elephants are there allowed to live in a semi-wild state.

Their mode of copulating is a subject which has excited considerable interest; also the age of maturity or period when they are capable of breeding.

It has generally been stated that the elephant is exceedingly modest, even more so than man; this, however, appears to be an erroneous statement. The only clear reason why they are not anxious to perform the act is that they are kept upon such low diet to keep them under control that they are, in reality, in a weak and debilitated condition, and their passions not easily aroused. When highly fed, however, they give every evidence of a strong desire to copulate, and when the female is in heat the power of man is utterly unable to restrain them.

The observations of Mr. Corse, who had a large number of elephants under his charge, was that they would copulate even when at work, in spite of all their drivers could do.

The record of his observations seem to be very accurate. First, he gives the record of an elephant born October 16th, 1789, which was a male, thirty-five inches high. The first year it increased in height eleven inches, measuring forty-six inches in height; the second year it gained only eight inches; the third, six inches; fourth, five inches; fifth, five inches; sixth, three and one-half inches; seventh, two and one-half inches, at that time measuring six feet and four inches in total height.

The males grow more rapidly than the females; but in one young female, which became pregnant at the age of sixteen years, in which actual measurements were recorded for five years previous to conception, the animal gained only six inches; but during the twenty-one months of her first pregnancy she gained five inches in height. Seven months later she again became pregnant, but during the second pregnancy gained only one-half inch. From this we must infer that the females gain slowly during their early years, but when they become pregnant at an early age rapidly increase in size.

Mr. Corse was inclined to think that the females became pregnant from the fifteenth year on, and that females, under natural circumstances, were fully matured by the nineteenth year.

Further than this, he had an opportunity of knowing accurately the days of copulation, and had the pleasure of following the case up to the day of delivery, the history of which is as follows: The bull elephant had connection with the female twice on the 28th and twice on the 29th of June, 1793, the four connections being within sixteen hours. The manner of copulation and the time occupied was the same as is commonly observed in the horse. Three months after connection the breasts began to enlarge, and continued gradually to increase until a few weeks before delivery, when their enlargement was more rapid. On March 16, 1795, she gave birth to a male, which measured thirty-five and one-half inches in height. This would fix the term of pregnancy at twenty months and eighteen or nineteen days. The infant sucked the milk from the breast with the mouth, often encircling the mammæ with its trunk, apparently to increase the flow.

In the early part of the following September, 1795, the animal was in heat, and readily took the male. At the end of a few days the female positively refused to take the male; and when he attempted to undertake liberties, he was treated to a kick in the face, and all attempts at copulation were stopped. As he left the station before she gave birth to the second elephant, he could not state positively the duration of the second gestation.

The signs of heat he states to be a slight swelling and congestion of the vulva, which also descended to a slight degree.

The measurements of the penis were given at two feet four inches to two feet six inches, and from fourteen to sixteen inches in circumference.

The reason given for their not breeding in captivity is that they are commonly kept on such low diet to render them docile and under perfect control; but as soon as they are highly fed, it is almost impossible to keep them from copulating and breeding.

Another interesting fact in connection with the elephant is that they are subject to a great variety of diseases. In India it is said that about fifty per cent. die within the first year of their captivity.

Again, he states that they often captured the female when carrying their young, and that the young, under such circumstances, are always smaller than those born in captivity, by some four or five inches. This fact, however, is ascribed to the depressing influence which the animals are subjected to gain perfect control over them.

The breeding of elephants in this country, however, is of the most recent date, and the only two have come under my personal observation, the first one being born at Philadelphia, Penn., on March 10th, 1880; the second at P. T. Barnum's Bridgeport winter quarters, at 8.10 o'clock P. M., February 2d, 1882.

Close observations were taken of the act of copulation, during pregnancy, and of the delivery.

The mother of the second is named Queen, and is between twenty-eight and thirty years of age.

The period of gestation was not quite as accurately determined as the one above quoted, as the bull was allowed access to her for several weeks, but dating from last known connection to the day of delivery, makes the period of gestation a few days less than twenty months. Conception may have followed the first act, which would increase the time a little.

For the first few months the animal appeared as usual, but as she advanced toward full term she grew heavy and sluggish until the last month, when she became decidedly lazy.

One of the earliest symptoms noted was the enlargement of the mammaæ, which in the elephant resemble the human both in general outline and position, being in the pectoral region.

From the fourth month the mammæ were quite prominent, but developed slowly until the last few weeks, when they rapidly gained in size until they were about as large as those of an ordinary Durham cow. As near as could be determined, each organ would measure from six to eight inches from base to point of nipple, and at the base about twenty-four inches in circumference.

Each nipple was covered until the day of delivery with a scaly coating.

On the morning of February 2, 1882, the animal appeared about as usual, but during the forenoon the scaly crusts over the nipples peeled off, and a watery mucous discharge was noticed coming from the numerous openings in each. Close observation revealed the fact that each nipple, instead of having a single or common opening, had several, the right eleven, and the left thirteen. About two hours before delivery there was a slight watery discharge from the vagina, and it was plain that the lips of the vulva were swollen, the vessels distended with blood, which appeared as so many blue lines.

When these symptoms were first noticed, the Queen was separated from the rest of the elephants, placed in a room by herself and securely fastened. She kept on eating, and seemed perfectly well until twenty minutes before the baby was born. From that time until birth she appeared a trifle uneasy, but gave no evidence of positive labor pains.

The delivery was very sudden, and occurred while she was standing, having previouly separated the posterior extremities to a slight degree.

The baby presented head and feet first, enclosed in its membranes, and appeared to be going up and out of the rectum, which appearance was accounted for by its passage over the pubic arch, and in less than three minutes had dropped to the ground. The mother instantly straightened up, crossed her posterior extremities, and by rubbing them together soon severed the cord. The little one lay perfectly quiet and apparently was not breathing, but the mother, as soon as the cord was broken, turned round, and with one of her anterior feet, struck the membranous sac quite forcibly, which instantly broke with a loud report. After rupturing the membranes, she placed her foot on the thorax, and pressed it with the appearance of considerable force, raised it and pressed again, and repeated this operation several times, until the little one began to breathe and gave positive evidence of life, when she ceased and appeared satisfied. Now, for the first time she was considerably excited. This stage of excitement lasted for about one-half hour. About this time the baby made several attempts to gain his feet, and finally succeeded, but was quite weak on his legs for a number of hours.

The mother gave every evidence of suffering more pain after the delivery and until the placenta was discharged, than in giving birth to the baby. There was a low beam near by, which she got astride of and settled down upon quite heavily until the after-birth was discharged, which occurred two hours after the fœtus. This was accompanied by a slight discharge of blood; the animal stepped back from over the beam, and appeared perfectly relieved.

Five hours after birth, the baby walked up to the mother, turned the trunk up over its head, and commenced nursing.

Two hours after delivery the baby was weighed, and turned the scale at one hundred and forty-five pounds, and measured thirty inches in height. The little one was of a light mouse-gray color, and has rapidly grown active and playful.

The milk soon became quite abundant, is nearly the same in color as that of the cow, but very sweet in taste, closely resembling that of the cocoanut milk. The quantity secreted daily is rather more than in the cow, and previous analyses have proven that it is much richer. The quantity of cream, as compared with the best Jersey cow that could be obtained, was found to be one-eighth greater in bulk.

DESCRIPTION OF ELEPHANT'S PLACENTA.

Oval in shape; longest diameter, from *a* to *b*, thirty-eight inches; shortest diameter, from *c* to *d*, twenty-two inches. *A*, placental tufts. *B*, endochorion. *C*, exochorion. *D*, umbilical cord. *E*, end of cord, showing cut extremity of vessels and encircling sheath. *F*, numerous cotyledons on the fan-shaped expansion of the chorion--in all about five hundred. *G*, vessels of the placental membranes.

An elaborate written description of the placenta is hardly necessary in connection with this case, as the above cut, with its explanatory notes, brings out clearly all the points of special interest.

A full account of the placenta taken from the elephant delivered in Philadelphia was reported upon by Henry C. Chapman, M.D., in the last volume of the Journal of the Academy of Sciences of Philadelphia, for the year of 1880.

The cord from the Bridgeport, Ct., case appeared like a large rope, and was formed by two arteries and one vein, and a remnant of the allantois, which were enclosed in a common sheath of dense fibrillated connective tissue substance. There was a small amount of adipose tissue surrounding the vessels and separating them from their ensheathing membrane.

The folds of the chorion were thickly studded with oval bodies which, in their gross appearance, closely resembled lymphatic glands. These structures were undoubtedly cotyledons, and numbered about five hundred in all. Two hundred were found to be located in the exochorion, and about the same number in the endochorion.

Microscopic sections of the vessels and cotyledons were prepared at the School of Histology and Pathology in connection with the Columbia Veterinary College, by William H. Porter, M.D., and Mr. William G. Le Boutillier, who found the following appearances: "The vessel, or umbilical vein, had its lumen nearly closed, which resembled in outline a small stellate opening. The tissue forming the wall of the vein was composed of non-striated muscle, white-fibrous and yellow-elastic tissue, arranged in concentric layers. The white-fibrous and elastic formed alternating layers, the latter being thin in comparison with the former, but at the same time very distinct. The muscular coat was thin and irregular. The endothelial layer not as well marked, although apparent.

"The two arteries also presented the star-shaped lumen, but their walls were made up principally of longitudinal and circular bands of smooth muscle fibres, the former predominating. These muscles were remarkably well developed.

"There was a thin external coat of white-fibrous and yellow-elastic tissue. There was also an imperfect tunica intima.

"Sections of the cotyledons showed that they were chiefly composed of mucous tissue containing numerous small thin-walled blood vessels, and all forms of connective tissue corpuscles, also a few involuntary muscle cells. The portions just within the circumference were more highly vascular, and were composed of a more dense or of a true fibrillated connective tissue substance. In the harder portions the blood vessels were less abundant and more perfectly formed. Around the outer edge of the section, or what would strictly represent the external layer or limiting membrane, there was a number of papillary projections composed of perfectly formed round and oval nucleated corpuscles, which would lead one to suppose that these nodules were covered in part at least by a layer of one or more very distinct cell elements."

---

See S. de Priezæ Hist. des Elephants, Paris, 1850.
Petrus ab Hartenfels, Elephant Tographia Curiosa, 17, 15.
Browning; Siam, its Kingdom and People, vol. 1, p. 219.
Livingston's Travels. Passim.
Philosophical Transactions of Royal Society, London, 1799.
Journal of Academy of Sciences, Philadelphia, 1880.

## Figures and Tables

**Figure f1:**